# Supplementary material for: VVX001 Induces preS-Specific Antibodies Reacting to Common HBV Genotypes in Hepatitis B Virus (HBV) Carrier Mice
Source: Vaccines (Basel). 2025 Aug 12;13(8):854. doi: 10.3390/vaccines13080854 (PMC12390538; doi:10.3390/vaccines13080854)
Supplement: Supplementary file 1 [file vaccines-13-00854-s001.zip › vaccines-3672277-supplementary.pdf]

Table S1. Sequences of HBV-derived synthetic peptides. Shown are the amino acid sequences of the peptides used in the assessment of preS-specific immune response.

preS-derived peptides	Accession №.	Sequence
preS peptide A, aa 1-29	AAT28735	**C**MGGWSSKPRKGMGTNLSVPNPLGFFPDHQ
preS peptide B, aa 30-61	AAT28735	**C**LDPAFGANSNNPDWDFNPIKDHWPAANQVGVG
preS A+B, genotype A, aa 17-51	AAT28735	**C**SVPNPLGFFPDHQLDPAFGANSNNPDWDFNPIKDH
preS genotype B, aa 13-51	BAA88276	GTNLSVPNPLGFFPDHQLDPAFKANSENPDWDLNPHKDN
preS genotype C, aa 13-51	BAA32833	GTNLSVPNPLGFFPDHQLDPAFGANSNNPDWDFNPNKDH
preS genotype D, aa 13-51	BAD02320	GQNLSTSNPLGFFPDHQLDPAFRANTANPDWDFNPNKDT
preS genotype E, aa 13-51	BAC65105	GKNHSTTNPLGFFPDHQLDPAFRANTRNPDWDHNPNKDH
preS genotype F, aa 13-51	AAG49720	GQNLSVPNPLGFFPDHQLDPLFRANSSSPDWDFNKNKDN
preS genotype G, aa 13-51	BAB64320	GKNLSTSNPLGFLPDHQLDPAFRANTNNPDWDFNPKKDP
preS genotype H, aa 13-51	BAB69786	GQNLSVPNPLGFFPDHQLDPLFRANSSSPDWDFNTNKDN

aa = amino acid

Table S2. Statistical evaluation of the preS-specific IgG_1 and IgG_{2a} levels. Shown are p values, significant differences are indicated: *p < 0.05, **p < 0.01.

**IgG_1 to preS**			
p-value at a time point	AAV-HBV + VVX001 vs. AAV-HBV + Vehicle	AAV-HBV + preS vs. AAV-HBV + Vehicle	AAV-HBV + VVX001 vs. AAV-HBV + preS
W0	0,7879	0,7879	0,7879
W5	0,8983	0,7879	> 0,9999
+3W	0,0022 **	0,0043 **	0,5714
+6W	0,0022 **	0,0022 **	0,3052
+9W	0,0022 **	0,0022 **	0,5714
+12W	0,0022 **	0,0022 **	> 0,9999
+14W	0,0022 **	0,0022 **	0,0931
p-value at a time point	AAV-Empty + VVX001 vs. AAV- Empty + Vehicle	AAV- Empty + preS vs. AAV- Empty + Vehicle	AAV- Empty + VVX001 vs. AAV- Empty + preS
W0	0,1797	0,3052	> 0,9999
W5	0,0411 *	0,5714	0,2381
+3W	0,0022 **	0,0022 **	0,4740
+6W	0,0022 **	0,0022 **	0,6753
+9W	0,0022 **	0,0022 **	0,5714
+12W	0,0022 **	0,0022 **	0,2381
+14W	0,0022 **	0,0022 **	0,5714
**IgG_{2a} to preS**			
p-value at a time point	AAV-HBV + VVX001 vs. AAV-HBV + Vehicle	AAV-HBV + preS vs. AAV-HBV + Vehicle	AAV-HBV + VVX001 vs. AAV-HBV + preS
W0	0,3874	0,5714	0,5714
W5	0,8983	> 0,9999	0,5714
+3W	0,0022 **	0,0260 *	0,7879
+6W	0,0022 **	0,0022 **	0,5714
+9W	0,0022 **	0,0022 **	0,1797
+12W	0,0022 **	0,0022 **	0,0260 *
+14W	0,0022 **	0,0022 **	0,0649
p-value at a time point	AAV-Empty + VVX001 vs. AAV- Empty + Vehicle	AAV- Empty + preS vs. AAV- Empty + Vehicle	AAV- Empty + VVX001 vs. AAV- Empty + preS
W0	0,3874	0,2381	0,0152 *
W5	0,4740	0,3052	0,7879 *
+3W	0,0022 **	0,0022 **	0,0260 *
+6W	0,0022 **	0,0022 **	0,0043 **
+9W	0,0152 *	0,0411 *	0,3874
+12W	0,0022 **	0,0022 **	0,0931
+14W	0,0022 **	0,0022 **	0,0931

Table S3. Endpoint titration of the preS-specific IgG_1 and IgG_{2a}.

OD_{405nm}	IgG_1	w5	+3w	+6w	+9w	+12w	+14w	IgG_{2a}	w5	+3w	+6w	+9w	+12w	+14w
AAV-HBV	1:100	0.185082	1.321697	1.299972	1.316276	1.2993	1.203371	1:50	0.044751	0.393794	0.514823	0.581446	0.867202	0.87371
VVX001	1:500	0.022583	0.496761	0.945213	1.035835	0.895799	0.785388	1:250	0.009947	0.135766	0.247334	0.344999	0.556501	0.620661
	1:1000	0.014551	0.289501	0.553858	0.605193	0.536921	0.506655	1:500	0.019952	0.075968	0.136522	0.178753	0.321034	0.321906
	1:1500	0.008789	0.206038	0.447463	0.474877	0.440246	0.386059	1:1000	0.009947	0.039206	0.081611	0.113313	0.164094	0.161767
	1:2000	0.006868	0.178042	0.371276	0.361148	0.350846	0.349333	1:1500	0.007329	0.022744	0.057354	0.071955	0.110114	0.1061
	1:4000	0.01263	0.102437	0.248759	0.274834	0.239272	0.243637	1:2000	0.017741	0.037635	0.053224	0.071664	0.094175	0.089115
	1:6000	0.005937	0.051509	0.150047	0.177169	0.161804	0.15156	1:4000	0.000465	0.00698	0.016055	0.024838	0.042172	0.036879
	1:8000	0.012223	0.036668	0.124729	0.156857	0.153248	0.130782	1:6000	0.005003	0.005759	0.009831	0.016636	0.024489	0.022977
	1:10000	0.032128	0.029916	0.116406	0.146904	0.128512	0.110178	1:8000	0.028386	0.002792	0.019894	0.016229	0.017974	0.026816
AAV-HBV	1:100	0.187575	1.20414	1.426363	1.354909	1.295793	1.478407	1:50	0.044731	0.404821	0.356442	0.376601	0.400085	0.411086
preS	1:500	0.024789	0.203951	0.82926	0.957344	0.692016	0.563052	1:250	0.015143	0.044393	0.183433	0.223003	0.219995	0.1658
	1:1000	0.017802	0.103606	0.426688	0.528483	0.431294	0.317338	1:500	0.010372	0.020537	0.112176	0.148738	0.135202	0.126697
	1:1500	0.015474	0.071675	0.32655	0.374057	0.322513	0.252856	1:1000	0.013069	0.014936	0.071517	0.099055	0.089564	0.074213
	1:2000	0.018527	0.058013	0.272729	0.30911	0.247267	0.224445	1:1500	0.012913	0.023441	0.05378	0.072502	0.064152	0.058292
	1:4000	0.011489	0.029498	0.151269	0.206798	0.163637	0.142109	1:2000	0.011098	0.025775	0.038429	0.040141	0.039155	0.055232
	1:6000	0.010764	0.0133	0.10019	0.16669	0.120994	0.089271	1:4000	0.008453	0.011565	0.039	0.058344	0.053728	0.030131
	1:8000	0.013455	0.012576	0.096723	0.127722	0.095843	0.07328	1:6000	0.011669	0.010891	0.019604	0.026346	0.02313	0.035214
	1:10000	0.031775	0.014801	0.105676	0.13269	0.083837	0.062567	1:8000	0.021056	0.011928	0.015143	0.026086	0.019811	0.022456
AAV-Empty	1:100	0.036384	1.299467	1.294153	1.152154	1.003401	1.087601	1:50	0.032697	0.226493	0.337462	0.482414	0.661104	0.854174
VVX001	1:500	0.011531	0.476895	0.957421	0.98961	0.81895	0.683669	1:250	0.016117	0.0847	0.224929	0.291203	0.535964	0.504032
	1:1000	0.009127	0.26546	0.604423	0.570419	0.482145	0.421595	1:500	0.016819	0.051613	0.144999	0.180696	0.324289	0.310181
	1:1500	0.008636	0.185527	0.481458	0.426158	0.408396	0.325863	1:1000	0.035848	0.022895	0.031329	0.032886	0.05081	0.052065
	1:2000	0.004956	0.144015	0.369533	0.350642	0.37709	0.322575	1:1500	0.01943	0.032183	0.085804	0.099059	0.165885	0.159509
	1:4000	0.010206	0.099609	0.286068	0.319484	0.279886	0.190483	1:2000	0.011799	0.027514	0.061253	0.076767	0.129736	0.107293
	1:6000	0.006183	0.05795	0.177038	0.170611	0.180522	0.16065	1:4000	0.020987	0.022192	0.046643	0.053672	0.092683	0.082692
	1:8000	0.008243	0.044848	0.147156	0.150934	0.161091	0.101081	1:6000	0.013707	0.012903	0.022945	0.024401	0.037153	0.034392
	1:10000	0.030717	0.039549	0.120315	0.127332	0.15854	0.1106	1:8000	0.044182	0.014359	0.018175	0.020033	0.027514	0.029924
AAV-Empty	1:100	0.047921	1.25204	1.228346	1.176202	1.166181	1.161973	1:50	0.034088	0.3564	0.420502	0.395168	0.40856	0.411273
preS	1:500	0.017855	0.539033	0.977602	0.953986	0.896802	0.693349	1:250	0	0.088203	0.220738	0.209182	0.234902	0.2371
	1:1000	0.017284	0.332628	0.685207	0.583552	0.60136	0.438664	1:500	0	0.046633	0.150585	0.13729	0.160913	0.158203
	1:1500	0.010856	0.248638	0.51975	0.445568	0.459519	0.387432	1:1000	0	0.018561	0.105588	0.07542	0.094186	0.088919
	1:2000	0.009761	0.181932	0.434664	0.387194	0.42638	0.357054	1:1500	0	0.006289	0.054916	0.043667	0.077721	0.064069
	1:4000	0.011522	0.09437	0.244496	0.256494	0.308631	0.220832	1:2000	0	0.028225	0.041264	0.038247	0.04469	0.042031
	1:6000	0.009237	0.070468	0.19812	0.169219	0.235925	0.175218	1:4000	0	0	0.010942	0.010022	0.016874	0.021936
	1:8000	0.009094	0.055517	0.186645	0.160315	0.170171	0.153078	1:6000	0.007823	0	0.001994	0.005318	0.007721	0.019635
	1:10000	0.033234	0.053232	0.153697	0.128509	0.152125	0.138889	1:8000	0.00767	0	0.005011	0.005267	0.017487	0.02664
AAV-HBV	1:100	0.200244	0.171791	0.135053	0.102034	0.076316	0.108178	1:50	0.063873	0.063701	0.05562	0.047376	0.046354	0.048658
Vehicle	1:500	0.024826	0.011363	0.011984	0.004392	0.004345	0.007448	1:250	0.026969	0.012415	0.009021	0.01646	0.017251	0.022691
	1:1000	0.011793	0.004583	0.002865	0.00148	0.003103	0.004249	1:500	0.028736	0.00916	0.007626	0.009811	0.013577	0.03571
	1:1500	0.013989	0.004488	0.00191	0.000764	0	0.004536	1:1000	0.024272	0.010183	0.007207	0.007114	0.015716	0.016042
	1:2000	0.009978	0.001289	0.003915	0	0.002483	0.003056	1:1500	0.021947	0.00916	0.008742	0.008649	0.013112	0.0239
	1:4000	0.00931	0.002339	0.007878	0.002817	0.002339	0.005968	1:2000	0.011671	0.016042	0.008928	0.016786	0.021436	0.026271
	1:6000	0.008164	0.003533	0.000239	0.00043	0.000907	0.002865	1:4000	0.015809	0.012601	0.005068	0.008277	0.008835	0.016693
	1:8000	0.005729	4.8E-05	0	0	0.001337	0.002196	1:6000	0.011485	0.0146	0.011624	0.007254	0.015902	0.015112
	1:10000	0.033707	0.001814	0.003819	0.0074	0.009931	0.0053	1:8000	0.019901	0.023156	0.006975	0.012554	0.031805	0.020738
AAV-Empty	1:100	0.042791	0.042117	0.039247	0.039395	0.039313	0.040218	1:50	0.045307	0.04961	0.050522	0.140751	0.093184	0.083339
Vehicle	1:500	0.014409	0	0	0	0.003212	0.013039	1:250	0.018718	0.017476	0.011798	0.013573	0.013573	0.020048
	1:1000	0.022629	0.001984	0	0	0	0.002173	1:500	0.031226	0.009448	0.01029	0.009181	0.008028	0.013617
	1:1500	0.009826	0.007748	0	0	0	0	1:1000	0.011754	0.009314	0.009891	0.006742	0.005544	0.014504
	1:2000	0.012897	0	0.00274	0	0	0	1:1500	0.017298	0.022177	0.012153	0.007806	0.017165	0.012109
	1:4000	0.016818	0.00274	0	0.000756	0	0.003212	1:2000	0.020669	0.032955	0.028609	0.023597	0.020891	0.029185
	1:6000	0.004488	0.007086	0	0	0.004819	0	1:4000	0.009891	0.011133	0.007939	0.005189	0.009093	0.011443
	1:8000	0.01044	0.00274	0.001323	0.005669	0	0.001701	1:6000	0.012198	0.00581	0.005367	0.009137	0.007052	0.011798
	1:10000	0.051588	0	0.00189	0.005858	0.001748	0.007417	1:8000	0.011266	0.006831	0.00479	0.022266	0.013661	0.010956

Cut-off for IgG_1: 0.144644456. Cut-off for IgG_{2a}: 0.168644298

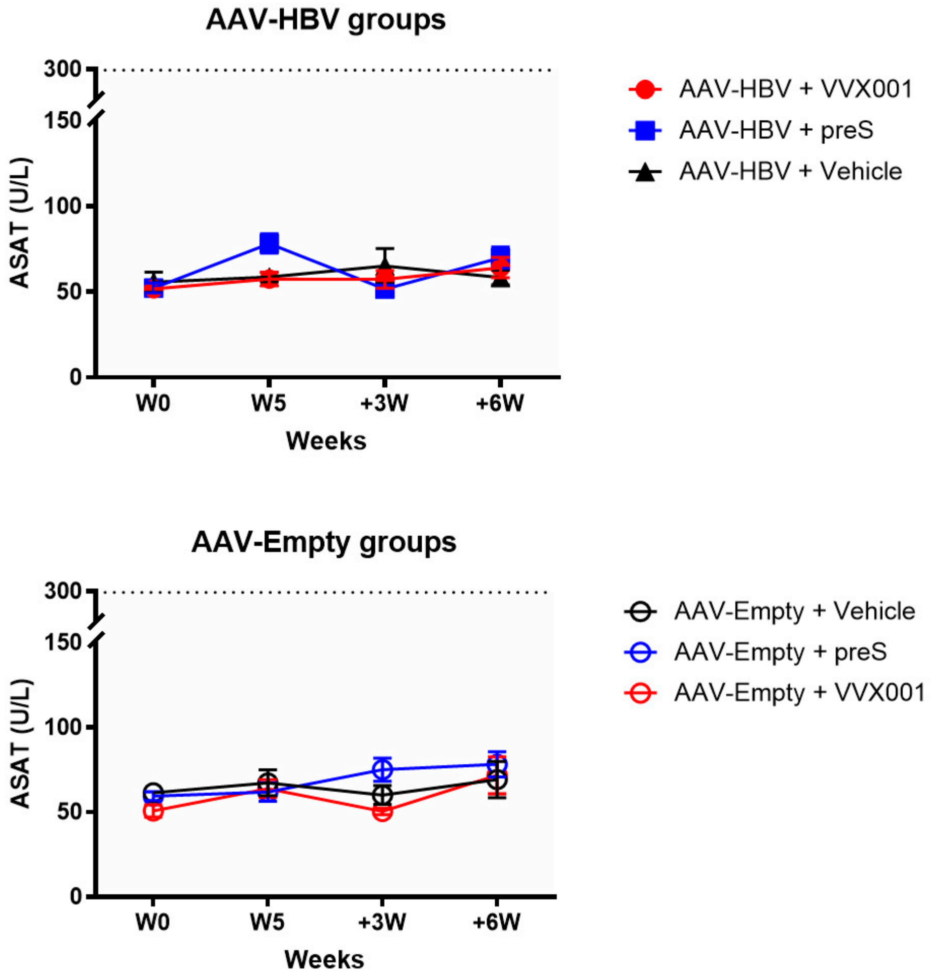

Figure S1. Aspartate aminotransferase activity during the experiments. ASAT activity was measured in AAV-HBV (upper panel) and in AAV-Empty (lower panel) groups. ASAT levels are given in international units per liter (IU/L) and are expressed as mean values ± SEM, a horizontal line indicates the upper limit of the normal ASAT level.
